# The Impact of Genetics on Cognition: Insights into Cognitive Disorders and Single Nucleotide Polymorphisms

**DOI:** 10.3390/jpm14020156

**Published:** 2024-01-30

**Authors:** Giulia Spoto, Gabriella Di Rosa, Antonio Gennaro Nicotera

**Affiliations:** 1 Unit of Child Neurology and Psychiatry, Department of Biomedical Sciences, Dental Sciences & Morphofunctional Imaging, University of Messina, 98125 Messina, Italy; giulia.spoto27@gmail.com; 2 Unit of Child Neurology and Psychiatry, Department of Human Pathology of the Adult and Developmental Age “Gaetano Barresi”, University of Messina, 98125 Messina, Italy; antonionicotera@ymail.com

**Keywords:** autism, COMT, executive functions, genetics, MTHFR, neurocognition, prefrontal cortex, schizophrenia

## Abstract

This article explores the complex relationship between genetics and cognition, specifically examining the impact of genetic variants, particularly single nucleotide polymorphisms (SNPs), on cognitive functions and the development of neuropsychiatric disorders. Focusing on neurotransmitter regulation within the prefrontal cortex’s dopaminergic circuits, this study emphasizes the role of genes like COMT, PRODH, and DRD in shaping executive functions and influencing conditions such as ADHD and schizophrenia. Additionally, it explores the significance of genetic factors in neurodevelopmental disorders, emphasizing the need for early identification to guide appropriate therapeutic interventions. This article also investigates polymorphisms in the transsulfuration pathway, revealing their association with cognitive impairment diseases. Computational analyses, including machine learning algorithms, are highlighted for their potential in predicting symptom severity in ADHD based on genetic variations. In conclusion, this article underscores the intricate interplay of genetic and environmental factors in shaping cognitive outcomes, providing valuable insights for tailored treatments and a more comprehensive understanding of neuropsychiatric conditions.

## 1. Introduction

Cognition is a higher function of the human brain that includes the processes (i.e., language, memory, attention, executive functions, complex reasoning, social abilities) involved in acquiring knowledge that has to be inferred from behavior [1,2,3]. A cognitive system dysfunction is described in most neuropsychiatric disorders, contributing to defining the disease and affecting its functional outcome [4].

In the last few decades, cognitive neuroscience has focused on unravelling the neural mechanisms underlying cognitive processes and researching possible specific treatments to ameliorate cognitive performances. Instrumental investigations, such as electroencephalography, functional magnetic resonance imaging, and positron emission tomography, allow researchers to explore the functional activity of brain regions and networks involved in general cognition and specific abilities. On a molecular level, cellular and animal studies are more suitable to analyze neurotransmitters, receptors, and their interaction in physiologic and pathologic conditions and characterize the modifications induced by possible therapeutic compounds [4].

However, cognition is influenced by both biological and environmental factors. Numerous studies in the literature address the topic of the genetic effect on cognitive abilities and the changes in gene expression during brain development and throughout life, suggesting that half of the variance in general cognition can be attributed to genetic factors [3].

In this article, we will focus on the crucial impact that genetic variants may exert on general cognition or specific executive functions, with special attention to SNPs involved in the dopaminergic network and the transsulfuration pathway.

## 2. Genetics, Neurotransmitters, and Cognitive Disorders

Several genes have been linked to conditions that manifest at an early age, frequently affecting the development of cognitive functions [5,6]. For instance, monogenic disorder involving neurotransmitter regulation can influence neuronal excitability and synaptic efficacy in numerous areas of the central nervous system [5,7]. Neurotransmitters are crucial for proliferation, migration and differentiation of the nervous cells, as well as for neurite outgrowth, axonal guidance and synaptogenesis [8]. Notably, an imbalance between excitatory and inhibitory inputs may lead to impaired information processing and it has been implicated in the etiology of autism spectrum disorder (ASD), schizophrenia, anxiety, epilepsy, and substance abuse [9]. In these neurodevelopmental disorders, excitatory/inhibitory neurotransmission may influence the connectivity at the synaptic level impacting synaptic plasticity; therefore, it has been proposed as a possible mechanism affecting the stability of functional networks and cognitive function [10]. Genetic mutations affecting neurotransmitter regulation have the potential to disrupt the activity of neuronal networks during brain development, resulting in cognitive disorders such as intellectual development disorder and ASD [5,7,11]. Particularly, the prefrontal cortex (PFC) and its dopaminergic circuits have been highly implicated in cognitive processes, such as working memory, response inhibition, planning, attention, cognitive flexibility, decision making, and self-monitoring [12]. PFC receives inputs from the local inhibitory interneurons and pyramidal cells as well as excitatory projections from other limbic structures, and it is regulated by a layer of neuromodulators including catecholamines, acetylcholine, and serotonin [13]. Glutamate, the most abundant amino acid in the nervous system, represents the primary excitatory neurotransmitter. By contrast, gamma-aminobutyric acid (GABA) constitutes the main inhibitory neurotransmitter in the adult mammalian brain [9]. Alterations in glutamatergic and GABAergic systems have been implicated in social behavior and brain functional connectivity, supporting the connection between glutamatergic dysregulation and functional dysconnectivity in ASD [10,14]. Several animal models of autism proved an elevated excitatory synaptic input in comparison to the inhibitory synaptic inputs [15,16]. A study in humans proved reduced concentrations of glutamate and glutamine in the pregenual anterior cingulate cortex have been linked to more severe communication deficit symptoms in individuals with ASD [17].

The regulation of glutamate and GABA receptors and transporters has been linked to conditions involving cognitive impairment [9,18]. The genes encoding the proteins related to glutamate and GABA receptors and transporters are among the common genetic variants associated with ASD [19]. A very recent study on a mouse model proved that glutamatergic projections from the prelimbic cortex to distinct brain areas play an important role in emotion: projections to the basolateral amygdaloid nucleus mediate pyramidal neuron hyperactivity and anxiety-like behaviors, while the ones to the dorsal striatum mediate fast-spiking interneurons’ hyperexcitability and medium spiny neurons’ inhibition, leading to ASD-like behaviors [20]. Experiments on animal models suggest that the abnormal interaction of the metabotropic glutamate receptor subtype 5 (mGluR5) with the N-methyl-D-aspartate (NMDA) receptor can contribute to certain cognitive phenotypes of the Fragile X Syndrome, a disorder caused by mutations of the FMR1 gene and characterized by intellectual disability and autistic features [21]. In fact, postsynaptic activation of mGluR5 triggers long-term depression (LTD) in the hippocampus of FMR1-KO mice, inducing the internalization of the α-amino-3-hydroxy-5-methyl-4-isoxazolepropionic acid (AMPA) and NMDA glutamate receptors [22]. In addition, mGluR5-mediated signaling was selectively altered in striatal neurons of SHANK3 knockout mice, a different animal model of ASD. These changes resulted in perturbed function at striatal synapses, abnormal brain morphology, aberrant structural connectivity, and autistic features. In this model, in vivo recordings confirmed a tonic hyperactivation of the cortico-striatal-thalamic circuit in mutants, which became hypoactive during social behavior, underlying the deficits in learning and behavioral symptoms [14]. In Fragile X Syndrome, a decreased expression of glutamate transporter-1 (GLT-1) and glutamate re-uptake has been reported, resulting in abnormal neuronal hyperexcitability [23]. This mechanism may lead to pathological repetitive behaviors that can be reduced through NMDA receptor inhibitors [16,24]. The active phosphorylated cAMP response element-binding protein (p-CREB) acts as an activator of astrocytic GLT-1 gene expression, preserving glutamate balance, maintaining synaptic plasticity and preventing excitotoxicity. In fact, it is crucial in dendrite formation, spine growth, neuronal plasticity and long-term memory formation. Moreover, null mice for the GABA transporter 1 (GAT1) showed an impairment in the long-term potentiation (LTP) and therefore in hippocampus-dependent memory and learning [25].

Interestingly, a very recent study tried to underpin the mechanisms linking genetic and behavioral changes in ASD individuals with brain structure abnormalities. The authors revealed greater differences in cortical thickness between autism and controls in the regions with greater gene expression of glutamatergic and GABAergic genes, suggesting a role for excitatory/inhibitory imbalance. In fact, they found that cortical thickness in these regions is higher in adolescent patients with ASD and in typically developed adults, supporting the hypothesis that excitatory/inhibitory imbalance affects the overall cortical thickness in patients with ASD [26].

The serotoninergic network also acts as a modulator of the excitatory/inhibitory balance and, through the 5H-T7 receptor, it enhances the NMDA receptor-mediated synaptic plasticity and enhances the GABAergic interneurons in the hippocampus, exerting a pro-cognitive effect [7].

Indeed, genetics is a fundamental tool to understand the basis of psychopathologies and to delineate genotype-phenotype correlations and cognitive endophenotypes [1]. This characterization allows us to tie the peculiar physical, clinical, and cognitive features to specific genetic conditions [27,28,29]. In certain genetic pathologies, the neuropsychological and behavioral features may not be the prominent traits during infancy, but they become predominant during adolescence and early adulthood [30]. For instance, Rett syndrome (RTT) is peculiarly characterized by normal neurodevelopment until 18–24 months, followed by a rapid regression of acquired skills, including motor and higher cognitive abilities [31]. It has been reported that GABAergic dysfunction is a critical mediator of altered cognition in RTT phenotypes, since it has been implicated in impaired learning, memory, and social behavior. In particular, MeCP2 deficiency in GABAergic neurons results in altered glutamic acid decarboxylase (GAD)1/2 expression and changes in neuronal GABA content. In addition, MeCP2 affects the brain-derived neurotrophic factor (BDNF), a neurotrophin implicated in brain development that has a crucial role in modifying synaptic connections and modulating hippocampal LTP [32,33]. Delineating such modifications between childhood and adulthood is fundamental in recognizing the biological mechanisms that regulate typical and atypical development [3]. In parallel, the early recognition of underlying conditions in cognitive defects is crucial to starting an accurate therapy and preventing more severe outcomes. In fact, understanding the mechanisms that are at the basis of a genetic disease may contribute in the choosing of an effective therapy with lower cognitive burden [34].

The PFC circuitry is also involved in 22q11.2 deletion syndrome, a chromosomal disorder characterized by a hemizygous deletion in the long arm of chromosome 22. As a matter of fact, this region includes numerous genes involved in cognitive functions (i.e., COMT, PRODH, RTN4R, and DGCR8) and the patients show a higher risk for developing a psychotic disorder across their lifespan. In addition, impaired social cognition is among the main features of the syndrome, severely impacting the general outcome of the disease [35].

The Catechol-O-methyltransferase (COMT), encoded by the COMT gene, is the main one responsible for extra-synaptic catabolism of the dopamine (DA) in the PFC and appears to play an essential role in the modulation of fronto-striatal networks [12,36]. On the contrary, the proline dehydrogenase (PRODH) gene encoded a proline oxidase responsible for converting proline into D-1-pyrroline-5-carboxylate. Genetic mutations that cause a reduced activity of this enzyme result in type I hyperprolinemia, an autosomal recessive disorder characterized by cognitive/behavioral disturbances and epilepsy [37]. Both COMT and PRODH mutations have been implicated in modulating cognitive functions and susceptibility to psychiatric manifestations: the reduction in COMT activity determines an increased availability of dopamine in the PFC, and the elevation in proline caused by the impairment of PRODH activity leads to an increased glutamatergic signaling, which in turn induces a subsequent release of DA in the PFC. Therefore, the interaction between COMT and PRODH genes can cause an augmentation in dopamine activity, predisposing the patient to psychosis and schizophrenia [38].

## 3. SNPs and Neurocognition

Aside from rare single-gene disorders, an exciting strand of cognitive neuroscience is focused on single nucleotide polymorphism (SNP)-based heritability, referring to the genetic influence of common variants in genes involved in cognitive abilities [3].

Specifically, polymorphisms in genes that regulate the PFC circuits have been investigated in relation to cognitive impairments. For instance, the association between the Val158Met SNP of the Catechol-O-methyltransferase (COMT) and cognitive abilities have been extensively studied [12,36]. Results showed that this SNP impacts general executive functions, working memory, inhibition, and episodic and semantic memory [12]. Moreover, the COMT gene, together with FMR1 and DCX (encoding for doublecortin, a protein involved in the migration of post-mitotic neurons and cortical layering in the developing brain), has been linked to the Wnt signaling, a pathway responsible for hippocampal neurogenesis and synapse formation and remodeling. In fact, the Wnt signaling pathway and its modulators have been related to ASD and intellectual development disorder [39]. In addition, the Val158Met COMT has been proven to modulate PFC-dependent responses to drugs, especially those that work on the dopaminergic system [36,40].

Also, the monoamine oxidase (MAO) is another important enzyme involved in the PFC circuitry, being responsible for the deamination of monoamine neurotransmitters. Notably, MAO-A is involved in the catabolism of serotonin; a recent study on its polymorphism MAOA-uVNTR showed that a high activity allele is related to lower 5-HT levels, leading to worse performance in social cognition [41]. Accordingly, this polymorphism has been reported in relation to enhanced aggressive behaviors in animals and humans, cause by increased amygdala reactivity and decreased prefrontal activity during emotional arousal [42,43]. More recently, the interaction between this gene and the polymorphism of serotonin transporter 5-HTTLPR has been studied in relation to the pre-attentive processing of threatening voices, proving an impact in male individuals on threat processing and social cognition [44].

Regarding the PFC, the SNPs of dopamine receptors (DRD) in the pyramidal neurons are also involved in cognition: the TaqIA DRD2 variant has been shown to influence verbal memory, learning, and visuospatial abilities, while the 4R variant of the 48 bp VNTR SNP in the DRD4 gene has been consistently related to poorer performances in attention, executive functioning, and inhibition, suggesting a crucial role in Attention Deficit/Hyperactivity Disorder (ADHD) [12]. Moreover, DRD2 polymorphisms have been associated with ADHD, ASD, and the overlap of these two conditions [45]. The Ser9Gly polymorphism in the DRD3 gene has also been extensively studied in association with ADHD, since it is widely expressed in the mesolimbic areas, the critical regions for cognitive and emotional functions, novelty seeking, and expression of reward. It has been demonstrated that the T allele carriers are less sensitive than the C allele carriers to the effects of DA, possibly showing a lower response to methylphenidate treatment [46].

Furthermore, the Val66Met SNP of the BDNF has been proven to impact memory and executive functions for its higher expression in the hippocampus: it affects monoaminergic neurotransmitter systems, increasing the serotonergic tone and influencing dopaminergic activity [12]. Recently, a study conducted on 18 individuals (9 Val homozygotes and 9 Met allele carriers) suggested that BDNF may participate in the excitatory/inhibitory imbalance of the motor cortex. According to this hypothesis, the BDNF genotype is related to the strength of excitatory and inhibitory neural circuitry. As a matter of fact, the BDNF Val66Met Val/Val homozygotes showed an increased response to LTP- and LTD-like forms of transcranial magnetic stimulation compared to Met allele carriers [47].

In addition to the genes related to the PFC, polymorphisms involved with the transsulfuration pathway have been associated with cognition. Pathogenic variants and SNPs of the methylenetetrahydrofolate reductase (MTHFR) gene have been consistently reported in diseases present with cognitive impairment, such as Alzheimer’s disease, Parkinson’s disease, epilepsy, and cerebrovascular lesions [48,49,50,51]. Although not always directly associated with alterations in cognitive abilities, polymorphisms of the MTHFR determine a reduced function of the enzyme, causing, in turn, increased levels of homocysteine, a well-known risk factor for cognitive impairment and brain development abnormalities [48,49,52]. Moreover, the His475Tyr polymorphism of the glutamate carboxypeptidase II (GCPII) has also been associated with cognitive abilities. GCPII is an enzyme that regulates folate absorption and neurotransmitters, influencing homocysteine levels [53]. The transsulfuration pathway is crucial in restoring the methionine supplies for epigenetic regulation and gene expression [48].

Finally, with the help of computational analysis, such as machine learning algorithms, SNPs of different genes (ADGRL3, DRD4, and SNAP25) have been used to predict symptoms severity in ADHD. In this study, polymorphisms of genes involved in dopamine circuitry (DRD4-rs916457), synaptic plasticity and working memory (SNAP25-rs362990), and hyperactivity and impulsivity symptoms (ADGRL3-rs2122642, ADGRL3-rs10001410) have been associated with the severity of ADHD under different genetic models of inheritance [54]. Another study performed a machine learning analysis to evaluate positron emission tomography imaging and genetic predictors involved in the serotonergic system. The authors propose that abnormalities in serotonergic transmission in ADHD may be depicted through an interplay between the striatum, insula and anterior cingulate cortex [55].

## 4. Conclusions

Although the effect of genetic factors is often subtle and difficult to interpret, the current literature indicates its important role in general and specific aspects of cognition. The intricate interplay between genetic factors and epigenetic regulation adds layers to this complexity. In fact, environmental factors may influence the experience-driven synaptic activity leading to long-lasting modifications of neural circuits and neuronal properties in the adult brain [5]. Mechanisms such as DNA methylation, chromatin remodeling/histone modifications, and microRNA regulation are responsible for the stability and accessibility to the chromatin, thus controlling gene expression [56]. Deciphering the pathological mechanisms that underlie neuropsychiatric disorders, along with unraveling the interaction between genetics and the environment, holds the key to tailoring targeted treatments. This intricate understanding promises to positively shape the prognosis of individuals facing these conditions.
